# Exendin-4 Induces Cell Adhesion and Differentiation and Counteracts the Invasive Potential of Human Neuroblastoma Cells

**DOI:** 10.1371/journal.pone.0071716

**Published:** 2013-08-22

**Authors:** Paola Luciani, Cristiana Deledda, Susanna Benvenuti, Roberta Squecco, Ilaria Cellai, Benedetta Fibbi, Ilaria Maddalena Marone, Corinna Giuliani, Giulia Modi, Fabio Francini, Gabriella Barbara Vannelli, Alessandro Peri

**Affiliations:** 1 Endocrine Unit, “Center for Research, Transfer and High Education on Chronic, Inflammatory, Degenerative and Neoplastic Disorders for the Development of Novel Therapies” (DENOThe), Department of Experimental and Clinical Biomedical Sciences, University of Florence, Florence, Italy; 2 Department of Physiological Sciences, University of Florence, Florence, Italy; 3 Department of Anatomy, Histology and Forensic Medicine, University of Florence, Florence, Italy; Dresden University of Technology, Germany

## Abstract

Exendin-4 is a molecule currently used, in its synthetic form exenatide, for the treatment of type 2 diabetes mellitus. Exendin-4 binds and activates the Glucagon-Like Peptide-1 Receptor (GLP-1R), thus inducing insulin release. More recently, additional biological properties have been associated to molecules that belong to the GLP-1 family. For instance, Peptide YY and Vasoactive Intestinal Peptide have been found to affect cell adhesion and migration and our previous data have shown a considerable actin cytoskeleton rearrangement after exendin-4 treatment. However, no data are currently available on the effects of exendin-4 on tumor cell motility. The aim of this study was to investigate the effects of this molecule on cell adhesion, differentiation and migration in two neuroblastoma cell lines, SH-SY5Y and SK-N-AS. We first demonstrated, by Extra Cellular Matrix cell adhesion arrays, that exendin-4 increased cell adhesion, in particular on a vitronectin substrate. Subsequently, we found that this molecule induced a more differentiated phenotype, as assessed by i) the evaluation of neurite-like protrusions in 3D cell cultures, ii) the analysis of the expression of neuronal markers and iii) electrophysiological studies. Furthermore, we demonstrated that exendin-4 reduced cell migration and counteracted anchorage-independent growth in neuroblastoma cells. Overall, these data indicate for the first time that exendin-4 may have anti-tumoral properties.

## Introduction

Glucagon-Like Peptide-1 (GLP-1) is mainly produced by enteroendocrine L-cells in response to nutrient ingestion and its principal effect is related to the induction of insulin secretion. Exendin-4 is a more stable GLP-1 analogue [1] currently used for the treatment of Type2 diabetes mellitus in its synthetic form exenatide. GLP-1 receptors (GLP-1R), mainly expressed in the pancreas, are also located in various organs and tissues including the central nervous system [2], where they regulate homeostatic functions, such as feeding behaviour, gastric motility, gluco-regulation and cardiovascular function [3]. GLP-1R knock-out mice present reduced learning abilities and are more susceptible to neuronal degeneration in the hippocampus than wild type mice [4] and *in vitro* neuroprotective effects of GLP-1 analogues have been thoroughly investigated [5], [6]. To date, little has been reported on the effects of GLP-1 and exendin-4 on tumor cells. GLP-1R expression is detectable in human tumors including endocrine tumors, tumors of the nervous system and embryonic tumors [7]. Recently, an inhibitory effect of exendin-4 on cell growth in colon CT26 [8] and in breast [9] cancer cells has been reported. The effect of molecules with neuroprotective and differentiating properties on tumor cell invasive potential has been investigated [10]. Moreover, the influence of gastrointestinal peptides belonging to the family of GLP-1 (e.g. Peptide YY and Vasoactive Intestinal Peptide) on cell adhesion and migration has been assessed in small intestinal cells [11] and in human T lymphocytes [12]. However, no data on the effects of exendin-4 on tumor cell motility are currently available. Studies addressing the pro-metastatic effect of Dipeptydil-Peptidase IV, the enzyme committed to the inactivation of GLP-1, on different types of cancer cells [13], [14], [15] suggest a possible role of exendin-4 on tumor cell motility. Neuroblastoma (NB) is the second most common solid tumor in children, metastatic in 70% of patients at diagnosis. NB arises from the developing sympathetic nervous system and its etiology is not clearly understood; metastatic spread of NB can happen by both lymphatic and hematogenous routes [16]. We have previously demonstrated differentiating actions of exendin-4 in NB SH-SY5Y cells, as assessed by the increasing number of neurites, changes in intracellular actin and tubulin distribution and increase of both Na^+^ channel conductance and Ca^2+^ currents (T- and L-type) amplitude, typical of a more mature neuronal phenotype [17]. In this study we investigate the effects of exendin-4 on cell adhesion, differentiation and migration, which in turn affects tumor spread and metastatization, in two NB cell lines and in human neuronal precursors, as a non-tumoral counterpart.

## Materials and Methods

### Cells and Treatments

The human NB cell lines SH-SY5Y and SK-N-AS (American Type Culture Collection, Manassas, VA, USA) were cultured in RPMI medium with 10% FBS, 2 mM L-glutamine, 100 IU/ml penicillin, 100 µg/ml streptomycin and maintained at 37°C in a humidified atmosphere (5% CO_2_/95% air). Fetal neuroepithelial cell cultures (FNC) were isolated from human fetal olfactory neuroepithelium by Vannelli *et al.* [18]. Reagents for cell cultures were from Sigma Chemical Co. (St. Louis, MO, USA). The cells were seeded in six-well plates, maintained in low serum conditions (RPMI medium with 2% FBS), and treated with exendin-4 (0.3 µM) (Sigma) for 24 and 48 h. The morphological evaluation of the differentiation of FNC and SK-N-AS cells was performed as previously reported [17]. Human mesenchymal stromal cells (hMSC) were isolated in collaboration with Dr. Riccardo Saccardi (Haematology Unit, Careggi Hospital, Florence, Italy), as described previously [19] and maintained for 24 hours in serum-free medium to obtain conditioned medium for migration assays in which we detected the release of stromal derived factor-1 (SDF1) by Quantikine ELISA Human CXCL12/SDF-1α Immunoassay (R&D systems). Cell proliferation was evaluated by cell counting and DNA synthesis [20], [21] and cell viability by MTS assay [17].

### Bengal Rose Adhesion Assay and ECM Cell Adhesion Array

Cells were seeded in a 96-well plate and treated with exendin-4 (0.3 µM) on the following day; after 24 or 48 h they were washed and incubated with bengal rose stain (0.25% in PBS, pH 7.3), then incubated for 30 minutes with an ethanol/PBS 1∶1 solution, allowing the adherent cells to release the stain, which was evaluated by an optical plate reader (Victor 3, Perkin-Elmer) at 560 nm. The interaction of the NB and FNC cells with different extracellular matrix (ECM) proteins was evaluated using an ECM cell adhesion array (Chemicon Int., Millipore, Billerica, MA, USA). Exendin-4 treated or untreated cells (10^6^ cells/ml) were non-enzymatically dissociated, resuspended in the assay buffer, seeded in a 96 well and incubated at 37°C for 5 h, allowing them to adhere to the protein-coated wells, and then washed. 100 µl of the cell stain solution was added for 5 minutes and then removed. The wells were washed and air-dried. Subsequently, the extraction buffer was added for 10 minutes and the absorbance was determined.

### 2D and 3D Matrigel Cultures

Matrigel (BD Bioscience, Bedford, MA) was thawed overnight on ice, diluted with cold serum free media (1∶3) and used both for the thin and thick gel methods. For the former, 50 µl Matrigel/cm^2^ of growth surface was used. The plates were allowed to sit for 30 minutes at 37°C and washed with serum free medium. Finally, cells were plated on top of the gel, with or without exendin-4, to evaluate cell morphology (2D cultures). For the latter, cells were added to Matrigel and suspended using cooled pipettes. Cell/Matrigel suspension (150 µl/cm^2^ of growth surface) was added and the plates were placed for 30 minutes at 37°C. Finally, growth medium was added to each well (3D cultures).

### Real-time RT-PCR

Total RNA was extracted using Nucleospin RNA II (Macherey Nagel) and reverse transcribed with TaqMan® Reverse Transcription Reagents (Applied Biosystems Inc., Foster City, CA). Primers and probe for uPAR (Hs00182181_m1), CXCR4 (Hs02330069_s1), microtubule-associated protein-2 MAP2 (Hs00159041_m1), Tau (Hs00902193_m1), Synaptophisin (Hs00300531_m1), Tissue inhibitor of metalloproteinases-1 (TIMP-1) (Hs99999139_m1), Matrix Metalloproteinase-9 (MMP-9) (Hs00957562_m1) were Assay-On-Demand products (Applied Biosystem). Primers and probe for human GLP-1R were R: 5′-GGCCAGCAGGCGTATTCA-3′ F: 5′-CCTCCTGCCACAGACTTGTTC-3′ probe: 5′ FAM-CAACCGGACCTT CG-TAMRA 3′. Each measurement was carried out in triplicate. The mRNA quantization was based on the comparative Ct (for cycle threshold) method and normalized to ribosomal 18S RNA expression.

### Electrophysiological Analysis

Patch pipettes (3–7 MΩ ) made as previously reported [17] were used for whole-cell current- and voltage-clamp recordings and filled with a solution containing (mM): 150 CsBr, 5 MgCl_2_, 10 EGTA and 10 HEPES. pH was 7.2, with KOH. Coverslips with the adherent cells (FNC after 48 h, and SK-N-AS up to 48 h) in culture, without (Control) and with exendin-4 were superfused as described previously [17]. Tetrodoxin (TTX) (1 µM) was used to test the voltage activated Na^+^ channels. To suppress K^+^ currents we made experiments in a 20 mM-TEA bath solution [17]. A Na^+^- and K^+^-free solution (Tetraethylammonium (TEA)-Ca^2+^ bath solution) was used to record Ca^2+^ currents [17]. To avoid the occurrence of the high voltage activated (HVAC) L-type Ca^2+^ currents, nifedipine (10 µM) was used; Cd^2+^ (0.8 mM) was used to block all the other HVACs. Ni^2+^ (50 µM) was used to block T-type Ca^2+^ currents [22]. Stretch activated channel (SAC) sensitivity was evaluated as reported previously [23], [17]. I_Na_ currents were recorded in cells bathed in 20 mM TEA solution with nifedipine by applying a pulse protocol from a HP of −80 mV with 10– ms step pulses ranging from –70 to 50 mV in 10-mV increments. I_Ca_ currents were elicited in TEA–Ca^2+^ solution with TTX added from a HP of −80 mV by 1-s depolarizing steps from −70 to 50 mV in 10-mV increments. The HP was held at −40 mV to inactivate most of I_Na_ and I_Ca_; a pulse protocol, 100 ms long, ranging from −80 to 0 mV in 10-mV increments was applied from a pre-step to −60 mV. The steady-state Na^+^ and Ca^2+^ currents activation was evaluated by
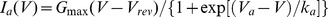
(1)


and the steady-state inactivation by
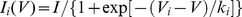
(2)where *G*_max_ is the maximal conductance for the *I_a_, V*_rev_ is the apparent reversal potential, *V*_a_ and *V*_i_ are the potentials eliciting the half-maximal current size, *k*_a_ and *k*_i_ are the steepness factors. Passive membrane capacitance, C_m_, and conductance, G_m_, were evaluated as reported previously [22]. The resting membrane potentials (RMP) were recorded by switching to the current clamp mode of the 200 B amplifier.

### Migration Assay

NB cell migration was measured in chemotaxis Boyden chambers. 13 mm PVP-free polycarbonate filters with 8 µm pores were coated using a Collagen type I solution (50 µg/ml in HCl 2 mM), air-dried and washed twice in serum-free RPMI medium with 0.2% bovine serum albumin (BSA). The lower compartment of the Boyden chamber was filled with 10% FBS RPMI or serum-free hMSC conditioned culture medium, the upper chamber contained the cell suspension (30000 cells/chamber in serum-free RPMI) treated or not with exendin-4. After incubation, the filters were removed and fixed with cold methanol for 30–40 minutes, then stained with the Diff-Quick kit (Dade Diagnostics of Puerto Rico Inc., Aquada, Puerto Rico) following the manufacturer’s instruction. The migrated cells were photographed with a phase-contrast microscope (Axiovert 25 Zeiss, NY, USA) at 400X magnification; 10 random fields were counted for each experimental point.

### Invasion Assay

The assay was performed in NB cells treated with exendin-4 (0.3 µM) by using the BD Matrigel Invasion Chamber kit (BD Biosciences, Bedford, MA), following the manufacturer’s instructions. The culture medium (RPMI with 10% FBS) was added to the wells of the plate as the chemotactic agent and immediately after the cell suspension was added to the inserts. At the end of the 22 h incubation, the non-migrated cells were removed, the migrated cells were fixed with cold methanol and stained using a Diff-Quick kit (Dade Diagnostics of Puerto Rico Inc) and then photographed at 200X magnification using a phase-contrast microscope AxioVision Zeiss (Zeiss Gottingen, Germany). The migrated cells were quantified considering 10 random fields per membrane.

### Soft-agar Colony Formation Assay

After assessing cell viability, 2×10^4^ cells were seeded in the presence of exendin-4 in triplicate in grid 60 mm dishes in 0.6% agar with 0.8% agar underlay. After 7, 14 and 21 days the plates were stained with 50 µg/ml MTT for 4 h at 37°C and the diameter of the colonies was measured. Experiments were repeated three times and for each experimental point, three plates were used.

### Statistical Analysis

Results are expressed as mean ± standard error (SE). Significance of differences (*p* values <0.05) between means was tested by one-way ANOVA with repeated measures followed by the appropriate post-hoc test.

## Results

### Effect of Exendin-4 on Cell Adhesion

**Figure 1:**
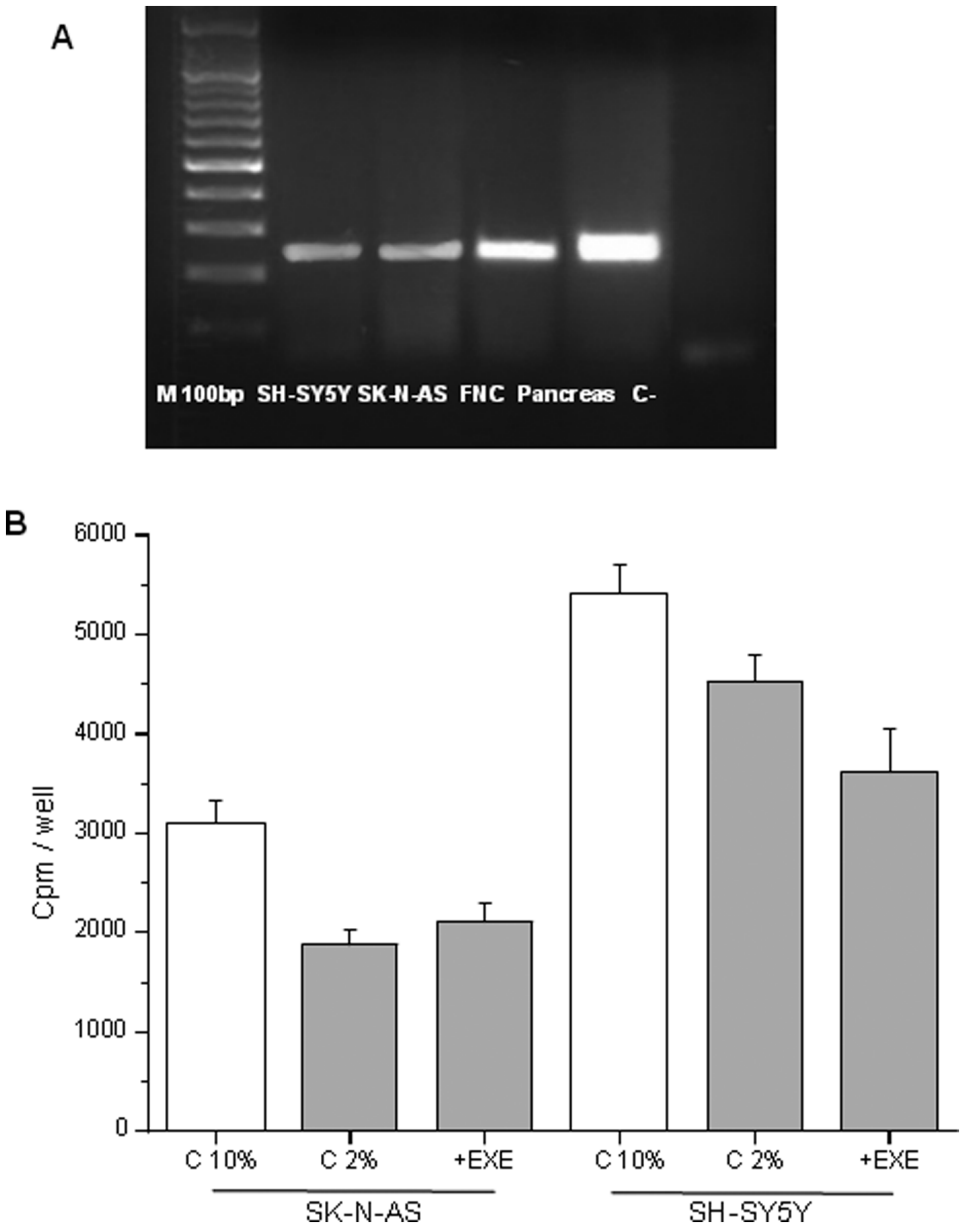
Expression of GLP1R and cell proliferation assays. RT-PCR analysis of the expression of the GLP1R in all the neuronal cell lines and in pancreas taken as the positive control. Amplicon length = 240 basepairs M 100 bp = Molecular weight marker 100 basepairs (A). Cell proliferation assay performed in SK-N-AS and SH-SY5Ycells after treatment with 0.3 µM exendin-4 (in 2% FBS) for 24 h. Cpm = counts per minute; C 2% = control cells in 2% FBS, EXE = exendin-4. +EXE *vs.* C 2%, p>0.05). C 10% = control cells in 10% FBS (standard medium, used as the positive control) (B).

**Figure 2:**
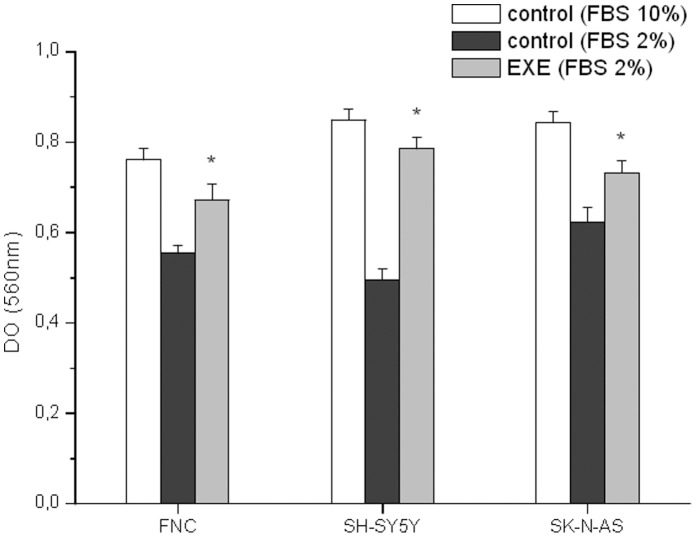
Cell adhesion assay. Effect of 24 h 0.3 µM exendin-4 treatment on cell adhesion as assessed by Bengal rose assay. Data are mean ± SE of three independent experiments. EXE = exendin-4. * = p<0.05 *vs* related control (FBS 2%); FBS 10% was the positive control.

**Figure 3:**
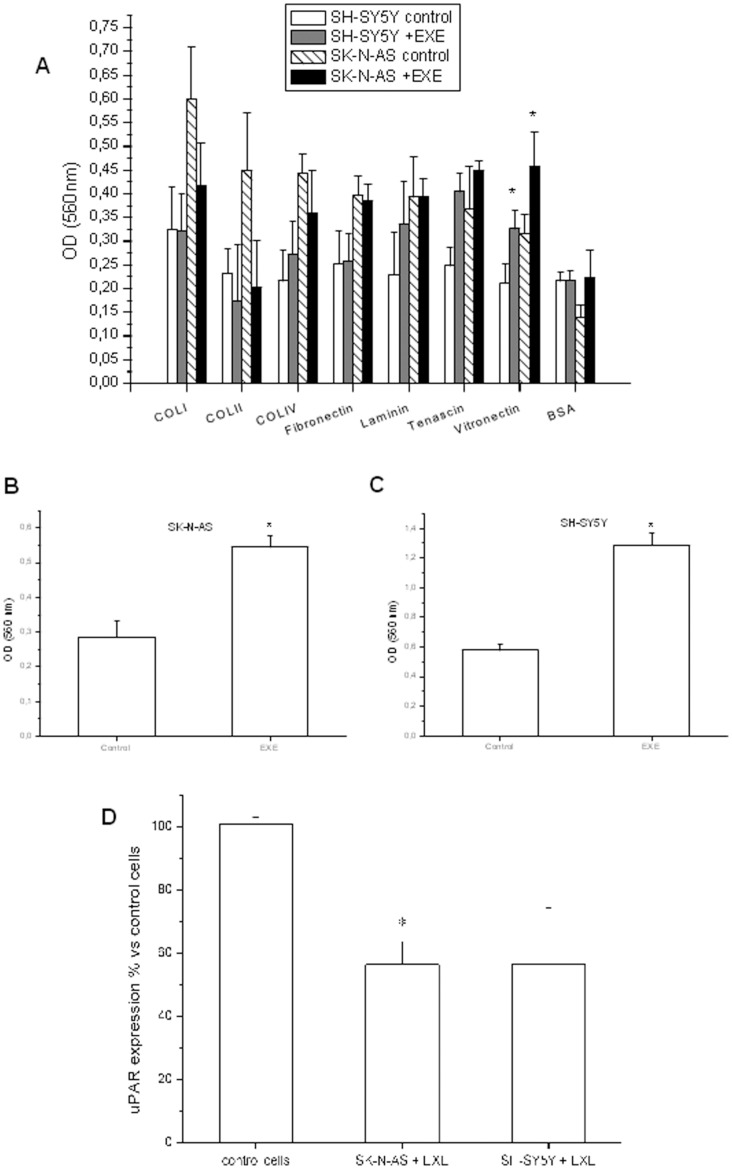
Cell adhesion assay on different ECM proteins and uPAR expression. Representative experiment on the effect of 0.3 µM exendin-4 on the adhesion of SH-SY5Y and SK-N-AS cells on different ECM proteins. * = p<0.05 *vs.* related control (A); Bengal rose adhesion assay performed on SK-N-AS (B) and SH-SY5Y (C) cells plated on vitronectin and treated with 0.3 µM exendin-4 for 24 h. A representative experiment of three independent experiments is shown in each case. * = p<0.05 *vs.* control; expression of uPAR as assessed by real-time RT-PCR in SK-N-AS and SH-SY5Y cells treated with 0.3 µM exendin-4 for 24 h. Mean percentage ± SE of four independent experiments. EXE = exendin-4. * = p<0.05 *vs.* control cells (D).

The presence of the GLP-1R in cells was evaluated by RT-PCR analysis (Fig. 1A). To test the effect of exendin-4 on cell proliferation and survival, we treated the cells with the GLP-1 receptor agonist exendin-4 at the dose of 0.3 µM. Exendin-4 did not significantly alter DNA synthesis (Fig. 1B), cell viability or cell counts (not shown). To demonstrate a possible effect of exendin-4 on cell adhesion, we performed a Bengal rose assay and we observed an increased number of adherent cells at 48 h in all the cells models (Fig. 2). In order to characterize which components of the Cell Adhesion Molecules (CAM) were involved in the increased adhesion properties, we screened the ability of the cells to adhere to different ECM proteins: collagen I, collagen II, collagen IV, fibronectin, laminin, tenascin, vitronectin. We found that both SH-SY5Y and SK-N-AS cells treated with exendin-4 augmented their interaction with vitronectin (Fig. 3A). Conversely, exendin-4 did not affect the adhesion of FNC to the proteins tested (Fig. S1A). To confirm data obtained with arrays, we performed cell adhesion tests in plates coated with vitronectin, and we found a significantly increased number of adherent NB cells (Fig. 3B and C), but not FNC cells (Fig. S1B). Moreover, since vitronectin is a major urokinase plasminogen activator surface receptor (uPAR) ligand with a role in cell adhesion, we measured the receptor expression by real-time RT-PCR, in order to evaluate whether exendin-4 increases the adhesion to vitronectin by up-regulating uPAR. As shown in figure 3D, exendin-4 did not increase uPAR expression in the two NB cell lines and actually mRNA levels appeared decreased (significantly in SK-N-AS), thus confirming that uPAR does not play a role in the stimulatory effect of exendin-4 on cell adhesion.

### Effects of Exendin-4 in 2D and 3D Culture Models

**Figure 4:**
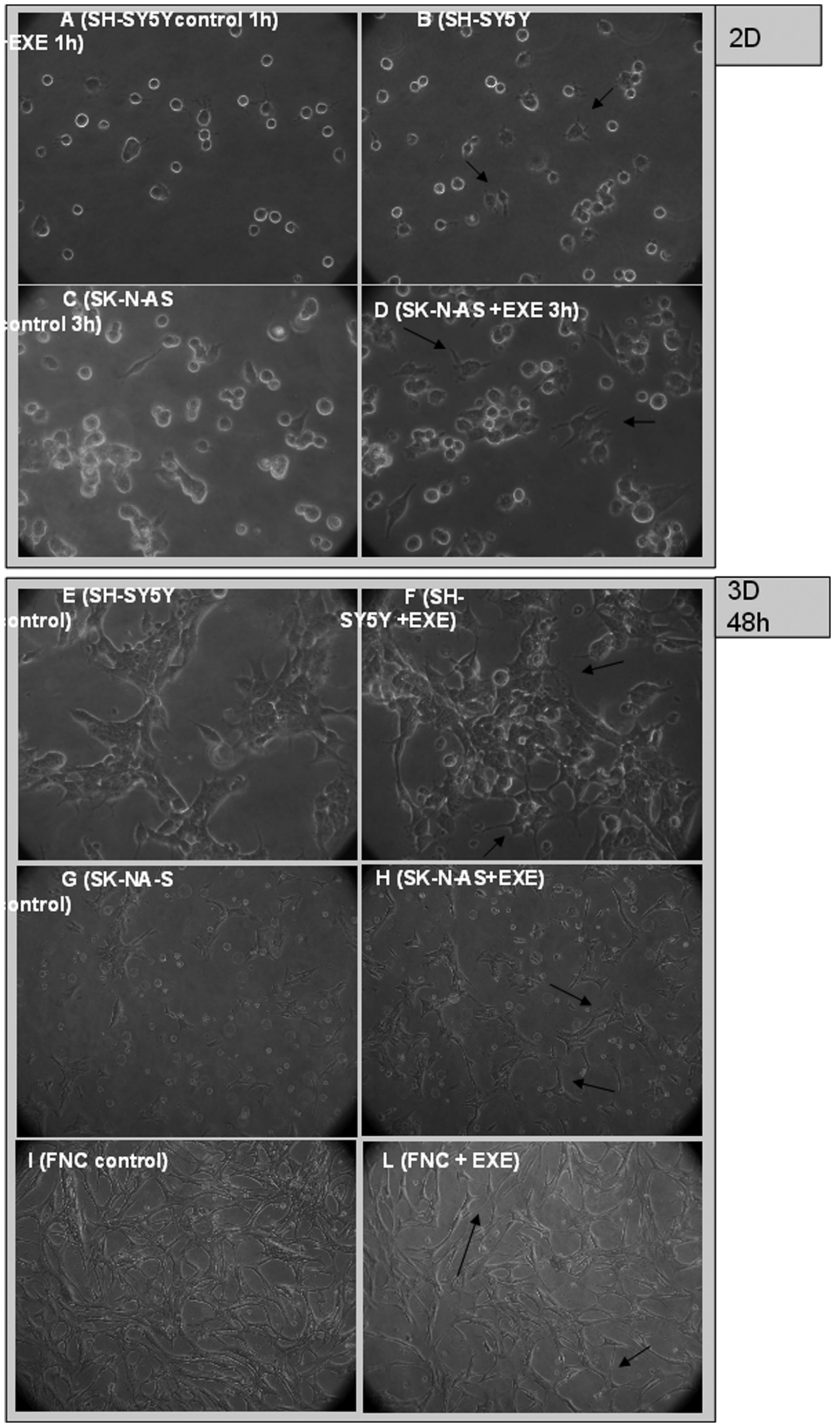
Effects of exendin-4 on SK-N-AS and SH-SY5Y in 2D or 3D matrigel cultures. Representative 400X phase-contrast inverted microscope fields of: SH-SY5Y control (A) and exendin-4-treated (B) cells after 1 h plating on top of matrigel; SK-N-AS control (C) and exendin-4-treated (D) cells after 3 h plating on top of matrigel: the arrows show the adherent cells in contrast to detached, rounded and refractile cells. Representative 100X phase-contrast inverted microscope fields of SH-SY5Y control (E) and exendin-4-treated (F) cells, SK-N-AS control (G) and exendin-4-treated (H) cells, FNC control (I) and exendin-4-treated (L) cells after plating inside matrigel for 48 h; long thin neuritic protrusions are indicated by the arrows and are suggestive of a more differentiated phenotype. EXE = exendin-4.

**Figure 5:**
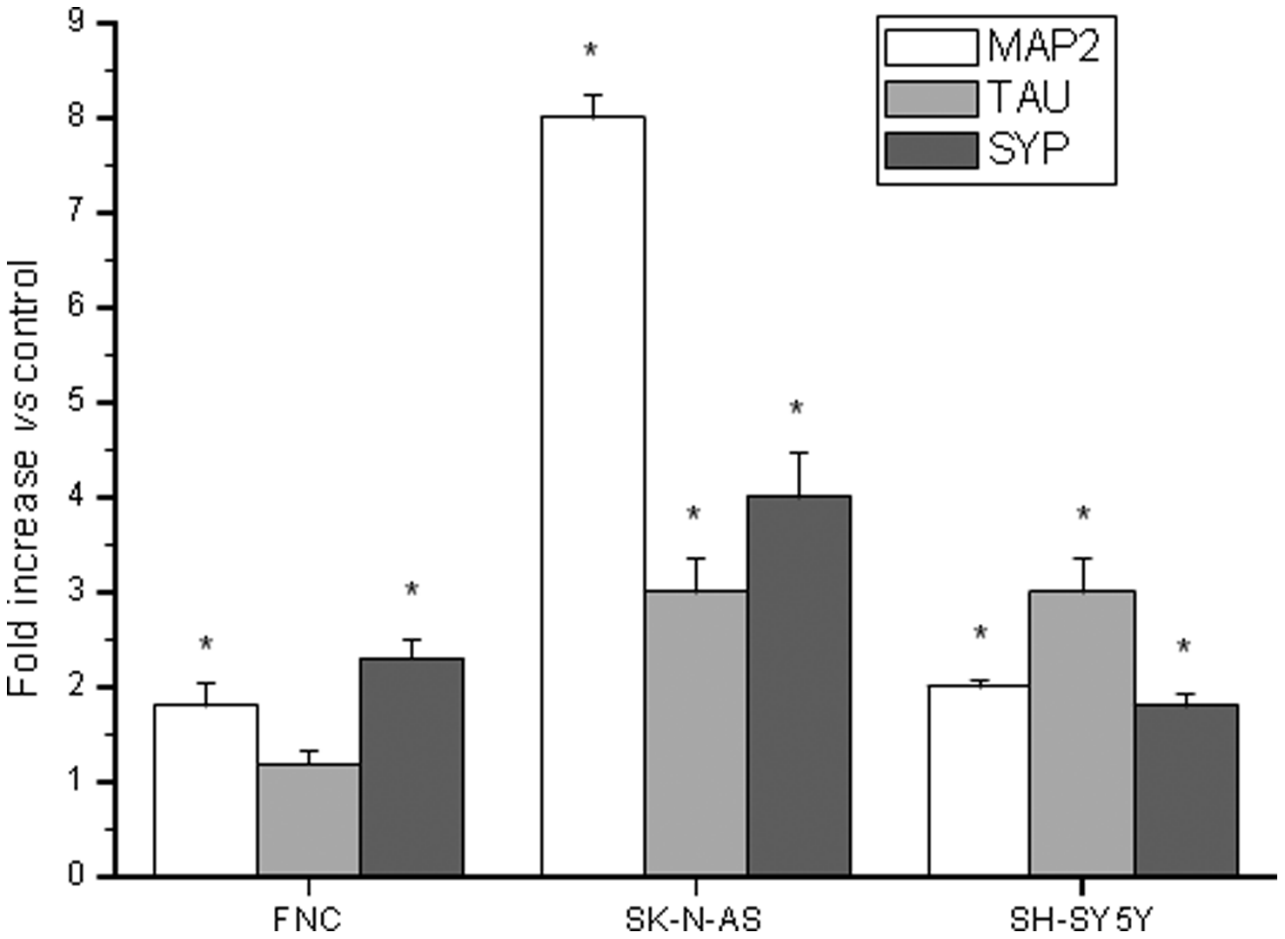
Neuronal markers expression. Expression of the neuronal markers MAP2, Tau and SYP after plating cells inside matrigel for 48 h, assessed by real-time RT-PCR. Results are expressed as mean fold increase ± SE *vs.* related control, taken as 1, of three independent experiments. * = p<0.05 *vs.* related control cells.

In vitro 3D cultures are relevant models of the cell-cell and cell-stroma interactions in both normal and cancer tissues [24] and allow the study of cellular differentiation, organization and migration. Using matrigel, we set up 2D cultures, plating cells on top, and 3D cultures, suspending cells inside the matrigel. We observed that soon after plating cells on top of matrigel, the number of cells spreading on the matrix was higher in samples exposed to exendin-4 than in controls (62±8% *vs.* 36±7% for SH-SY5Y after 1 hour p<0.05; 55±6% *vs.* 28±5% for SK-N-AS after 3 hours, p<0.05) (Fig. 4 A–D). No modification in adhesive properties was detected in FNC cells cultured on top of matrigel (Fig. S2). Furthermore, exendin-4-treated SH-SY5Y, SK-N-AS and FNC cells suspended inside the matrigel showed a rapid adaptation to the 3D environment, resulting in a more evident presence of neurite-like protrusions *vs.* control cells (Fig. 4 E–L). A similar effect had been already reported on plastic support for exendin-4-treated SH-SY5Y [17], whereas no effect was observed in SK-N-AS or FNC cells (not shown). We measured the expression of the neuronal markers MAP2, Tau and Synaptophysin; a significant increase of the transcripts in the cell lines tested was found, with the only exception of Tau in FNC (Fig. 5).

### Effects of Exendin-4 on the Membrane Passive Properties

**Figure 6:**
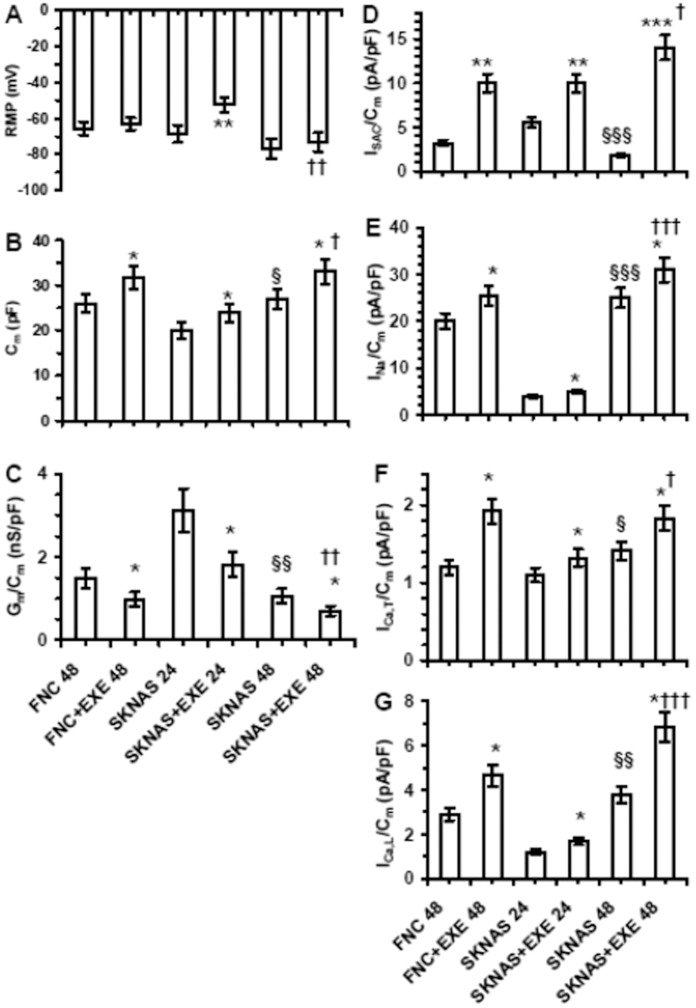
Analysis of passive properties and ionic current densities in FNC and SK-N-AS cells. Data were evaluated until 48 h in culture. Effect of Exendin-4 (EXE) on RMP (A), on membrane capacitance, C_m_ (B), and resting specific membrane conductance G_m_/C_m_ (C). * p<0.05 EXE- treated cells *vs.* the related control; § and §§ p<0.05 and <0.01 SK-N-AS 48 h *vs.* 24 h; †<0.05 and †† <0.01 SK-N-AS+Exendin-4 48 h *vs.* 24 h. FNC at 48 h, SK-N-AS at 24 and 48 h data are from 26, 28 and 32 cells, respectively. Effect of EXE on I_SAC_/C_m_ (D), I_Na_/C_m_ (E), I_Ca,T_/C_m_ (F) and I_Ca,L_/C_m_ (G). * and ** p<0.05 and P<0.01 EXE treated cells *vs.* the related control; §, §§ and §§§ p<0.05, <0.01 and <0.001 SK-N-AS 48 h *vs.* 24 h; †<0.05, †† <0.01 and ††† 0.001 SKNAS +EXE 48 h *vs.* 24 h. I_SAC_/C_m_ I_Na_/C_m_, I_Ca,T_/C_m_ and I_Ca,L_/C_m_ in FNC at 48 h, SK-N-AS at 24 and 48 h data in each experimental condition are from 14–18 cells.

When recorded in physiological bath solution the resting membrane potential (RMP) was similar in both control and exendin-4 treated cell models at 48 h, whereas exendin-4-treated SK-N-AS cells appeared more depolarized at 24 h (Fig. 6A). The cell-surface area evaluated by the membrane capacitance (C_m_) was significantly higher (Fig. 6B), whereas the specific resting membrane conductance (G_m_/C_m_) was reduced in both exendin-4-treated SK-N-AS and FNC (Fig. 6C). Such a reduction of G_m_/C_m_ is in agreement with diminished membrane leak currents and is an index of cell differentiation induced by exendin-4. Accordingly, the G_m_/C_m_ values in SK-N-AS at 24 h were greater than those recorded at 48 h in both control and exendin-4-treated cells. The results obtained in SK-N-AS at 48 h were similar to those observed in SH- SY5Y at the same time, as previously reported [17].

### Effects of Exendin-4 on the Current Density of Stretch-activated Channel in FNC and SK-N-AS Cells

Actin polymerization and its contractile status increase plasma membrane stiffness [25] and in turn increase the I_SAC_/C_m_ [23]. Moreover, we have demonstrated that exendin-4 treatment in SH-SY5Y cells dramatically increased F-actin accumulation and potentiated I_SAC_/C_m_ [17]. Here, we evaluated the action of exendin-4 on this latter parameter in the other neuronal cell model, i.e. SK-N-AS, and in FNC. The results clearly indicate that exendin-4 dramatically increased I_SAC_/C_m_ in both FNC and SK-N-AS. Interestingly, untreated SK-N-AS cells at 24 h showed a greater I_SAC_/C_m_ than at 48 h (Fig. 6D), and reduced size of I_Na_/C_m_ (Fig. 6E) and I_Ca,L_/C_m_ (Fig. 6G) whereas were less potentiated by exendin-4 treatment (Fig. 6D). Again, these findings confirm the more differentiated state at 48 h than at 24 h.

### Effects of Exendin-4 on Voltage-dependent Ionic Channels in FNC and SK-N-AS Cells

**Figure 7:**
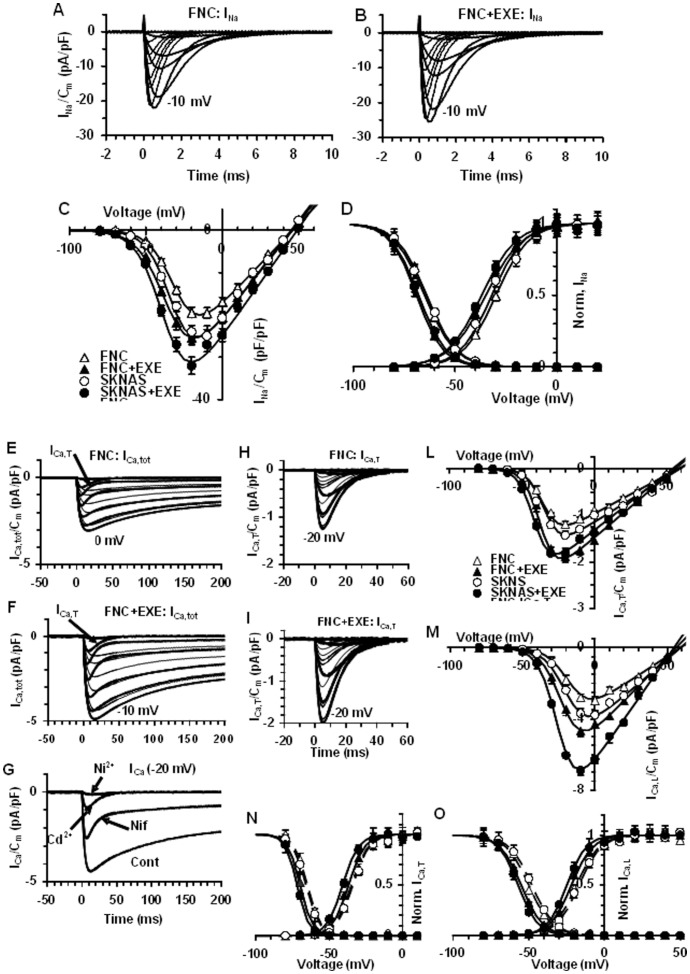
Analysis of voltage-dependent Na^+^ and Ca^2+^ channels in FNC and SK-N-AS cells. Typical I_Na_ traces recorded in a SK-N-AS cell. The voltage threshold of I_Na_ was at −50 mV (A). Effect of Exendin-4 (EXE) on I_Na_ amplitude (B). In A, B numbers represent the voltages eliciting the maximal I_Na_. C) Normalized I–V plots represent the data evaluated at the current peak in all the cells investigated; the Boltzmann fits (Eq. 1) are superimposed to the experimental data. D) Normalized data related to I_Na_ activation and inactivation and superimposed Boltzmann fit in control SK-N-AS and under exendin-4 treatment; the Boltzmann curves for activation are determined from panel C by the equation: 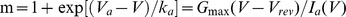 and inactivation from eq. 2; Boltzmann parameters listed in Table 1. Data represent mean ± SE from 26–43 cells. Representative I_Ca,tot_ traces obtained in a control (E) and in exendin-4 treated FNC cell (F). The arrow in the −50 mV trace indicates the presence of a first component as a fast-activating current, I_Ca,T_. High-voltage-activated and slowly inactivating current (I_Ca,L_, HVA) as a second component starting from −40 mV. Ca^2+^ currents elicited by a voltage step at −20 mV without (Cont) and in the presence of nifedipine (Nif), Cd^2+^ and Ni^2+^(G). Representative I_Ca,T_ recorded at a holding potential of –50 mV without (H), and with exendin-4 (I). Normalized I–V plots determined at the current peaks in control and under exendin-4 treatment related to I_Ca,T_ (L) and I_Ca,L_ (M). Normalized activation and inactivation data for T- (N) and L-type Ca^2+^ current (O) in control and under exendin-4 treatment, with the related Boltzmann fit superimposed to the data. The related Boltzmann parameters are listed in Table 1. In each experimental condition, data are from 18 to 23 cells.

In 20-mM TEA solution, untreated cells at 48 h exhibited INa, as shown in a typical experiment performed with FNC (Fig. 7A). The voltage threshold of INa was about −60 mV. The maximal INa was observed at −15 mV and was greater in SK-N-AS than in FNC. The treatment with exendin-4 increased INa amplitude (1.2 and 1.2 fold in SK-N-AS and FNC) (Fig. 7A, B and 6E). The normalized I–V plot is shown in Fig. 7C. The maximal current amplitude was elicited at −15±5 mV in control SK-N-AS and FNC cells, but it was shifted at −20±5 mV in exendin-4 -treated cells (Fig. 7C). Exendin-4 treatment changed the activation and inactivation data (Fig. 7D). In particular, the maximal current to peak (INa/Cm) and Gmax/Cm values increased to a similar extent than control, suggesting that the rise in current density was related to the channel conductance increase. The half voltage activation and inactivation values, Va and Vi, obtained by the Boltzmann fit, were shifted towards more negative potentials, of about 5 and 3 mV (SK-N-AS) and 7 and 4 mV (FNC), respectively. In contrast, ka values were unchanged, whereas ki diminished (Fig. 7D; Table 1). Therefore, we suggest that exendin-4 improved the INa occurrence both by significantly shifting Va and Vi and by enhancing Na+ channels conductance. The expression of functional Ca2+ channels was assessed in TEA-Ca2+ bath solution (Fig. 7E–O). In both cell types Ca2+ currents showed a low-voltage-activated and inward transient current (T-type Ca2+ current, ICa,T) with a voltage threshold at −50 mV, and a high-voltage-activated current with a slow inactivation (IHVA), which became evident from −40 mV. The fitting procedure to the activation and inactivation curves of these two currents resulted in two Boltzmann terms that were in agreement with T- and HVAC currents (Fig. 7 N–O). To verify this suggestion we added the L-type Ca2+ blockers Cd2+ or nifedipine to the bath. In our records T-type current was not affected by both molecules (Fig. 7H–I), whereas HVAC was blocked by Cd2+ and only partly by nifedipine. In fact, the current traces still showed a HVAC having amplitude of about the 10% of the control. Consequently, we can reasonably suppose that HVAC currents consisted of a large nifedipine-sensitive L- type current with a small fraction of other HVAC Ca^2+^ currents superimposed, most likely N, P, Q or R- types (Fig. 7G). Exendin-4 significantly increased I_Ca,T_, and I_Ca,L_, amplitude (Fig. 6 F–G). The normalized I–V plots and the related normalized Boltzmann curve related to T- and L-type current are shown in figure 7 L–M. Again, changes similar to those observed for I_Na_ were induced by exendin-4 in Ca^2+^ currents, such as an increase in G_max,Ca,T_/C_m_ and G_max,Ca,L_/C_m_, a shift towards a more negative potential of V_a_ and V_i_ and a decrease of k_a_ and k_i_. Notably, both the increase of the maximal current amplitude and the conductance of T- and L-type Ca^2+^ currents were greater than those of I_Na_, and the highest increases were those related to L-type Ca^2+^ current (Table 1). Again, the results obtained in SK-N-AS at 48 h were similar to those observed in SH-SY5Y at the same time as previously reported [17].

**Table 1:** Boltzmann parameters of activation and inactivation curves for I_Na_ and T- and L-type Ca^2+^ currents.

	I_p_/I_p,cont_	G/G_con_	V_a_ (mV)	k_a_ (mV)	V_i_ (mV)	k_i_ (mV)
I_Na_						
FNC (48 h)	1.0±0.1	1±0.1	−30.1±2	8.0±0.6	−65.1±5	7.5±0.7
FNC+EXE (48 h)	1.27±0.1 **	1.2±0.1 *	−35.4±3 *	9.0±0.8 *	−68.2±6	6.8±0.6
SKNAS (24 h)	1.0±0.1	1.0±0.1	−25±2	8±0.7	−68.1±6	6.9±0.5
SKNAS+EXE (24 h)	1.26±0.1 **	1.2±0.1 **	−30±3 *	7.8±0.6	−71.8±7	6.9±0.6
SKNAS (48 h)	1.0±0.1	1.0±0.1	−32.3±3	7.8±0.6	−64.2±6	7.4±0.7
SKNAS+EXE (48 h)	1.24±0.1 **	1.33±0.1 *	−37.1±3 *	8.5±0.7 *	−6.9±6	6.6±0.6 *
I_Ca,T_						
FNC (48 h)	1.0±0.1	1.0±0.1	−34.5±2	5.7±0.4	−64.8±5	4.5±0.2
FNC+EXE (48 h)	1.6±0.2 **	1.1±0.1 *	−37.6±3 *	5.5±0.3	−70.4±6 *	4.2±0.3
SKNAS (24 h)	1.0±0.1	1.0±0.1	−25±2	4±0.2	−60.0±6	4.9±0.2
SKNAS+EXE (24 h)	1.2±0.1 *	1.2±0.1 *	−30±3 *	4±0.2	−67.1±6 *	4.9±0.3
SKNAS (48 h)	1.0±0.1	1.0±0.1	−34±3	5.6±0.5	−65.7±6	4.6±0.3
SKNAS+EXE (48 h)	1.3±0.1 *	1.2±0.1 *	−41.0±4 *	5.4±0.6	−72.2±6 *	4.0±0.3 *
I_Ca,L_						
FNC (48 h)	1.0±0.1	1.0±0.1	17±2	7.4±0.6	−50.0±5	7.5±0.6
FNC+EXE (48 h)	1.6±0.2 **	1.2±0.1 *	−22±2 **	7.0±0.2	−55.2±5 *	6.9±0.6
SKNAS (24 h)	1.0±0.1	1.0±0.1	−16±1	7.4±0.8	−45.1±4	6.9±0.5
SKNAS+EXE (24 h)	1.4±0.2 **	1.2±0.1 *	−20±2 **	7.4±0.5	−47.7±4	6.9±0.6
SKNAS (48 h)	1.0±0.1	1.0±0.1	−18.7±2	7.5±0.6	−47.6±5 **	7.5±0.7
SKNAS+EXE (48 h)	1.8±0.2 ***	1.3±0.1*	−25.0±2 ***	6.4±0.6 *	−56.6±5 **	6.5±0.5 *

Effect of exendin-4 treatment on Boltzmann parameters of activation and inactivation curves for I_Na_ and T- and L-type Ca^2+^ current in FNC and SK-N-AS cells. * = p<0.05, ** = p<0.01 and *** = p<0.001 *vs*. the related control. I_p_/I_p_, _cont_ and G/G_cont_, represent the ratio of the peak currents and conductance in exendin-4 treated cells respect to their corresponding control. V_a_ and V_i_ are the half-voltages of activation and inactivation, respectively. k_a_ and k_i_ are the steepness factors for the activation and inactivation Boltzmann curve. EXE = exendin-4.

### Effect of Exendin-4 on Cell Migration

**Figure 8:**
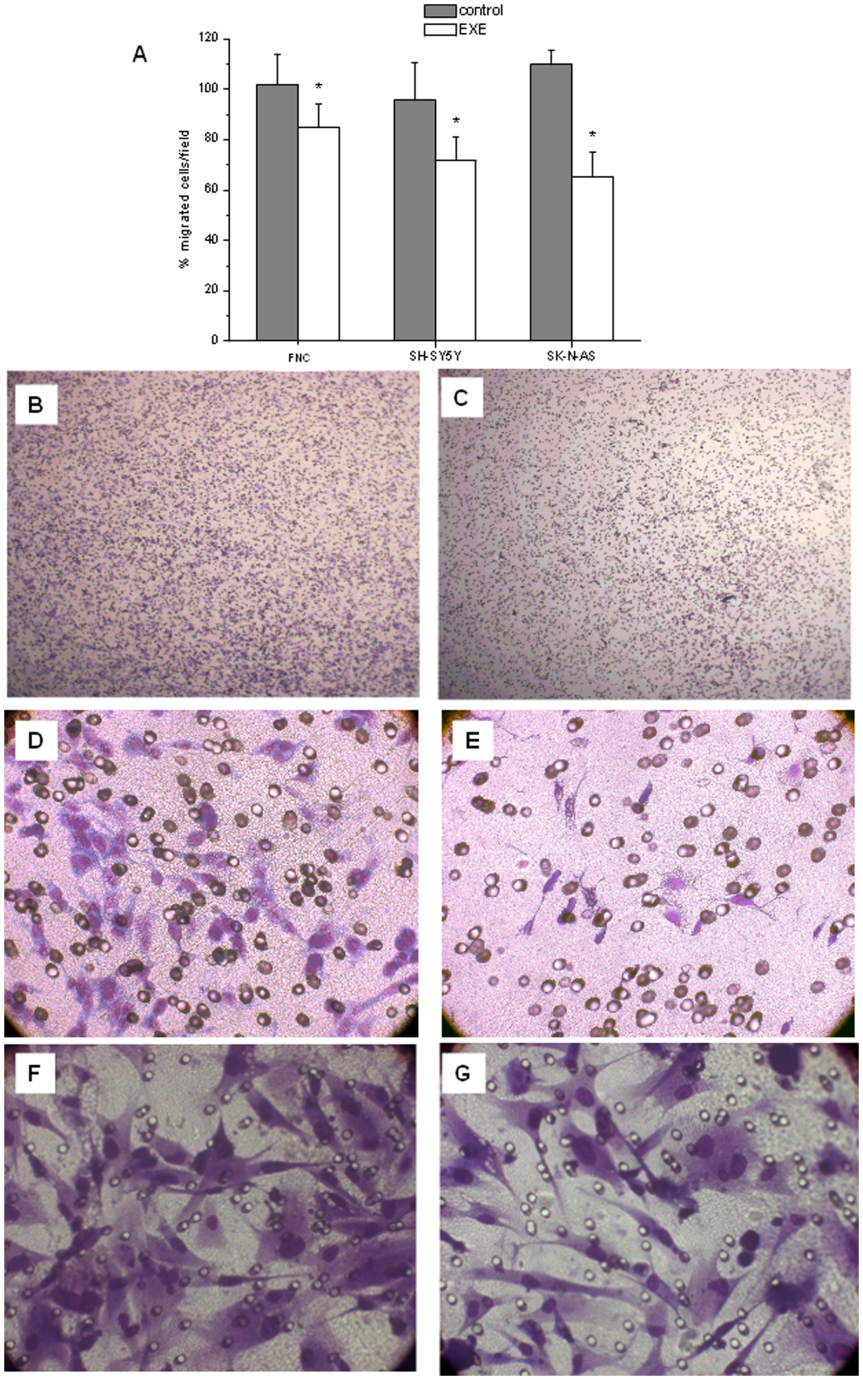
Migration assay. Effect of 0.3 µM exendin-4 (EXE) on FBS-induced migration of cells as assessed by Boyden chambers migration assay. Results are reported as mean percentage of migrated cells/field ± SE of four independent experiments, considering at least 10 random fields for each experimental point. * = p<0.05 *vs.* control (A); representative phase contrast inverted microscope pictures of migrated SH-SY5Y control (B) or exendin-4-treated (C) cells (50X magnification); SK-N-AS control (D) or exendin-4-treated (E) cells; FNC control (F) or exendin-4-treated (G) cells (400X magnification).

**Figure 9:**
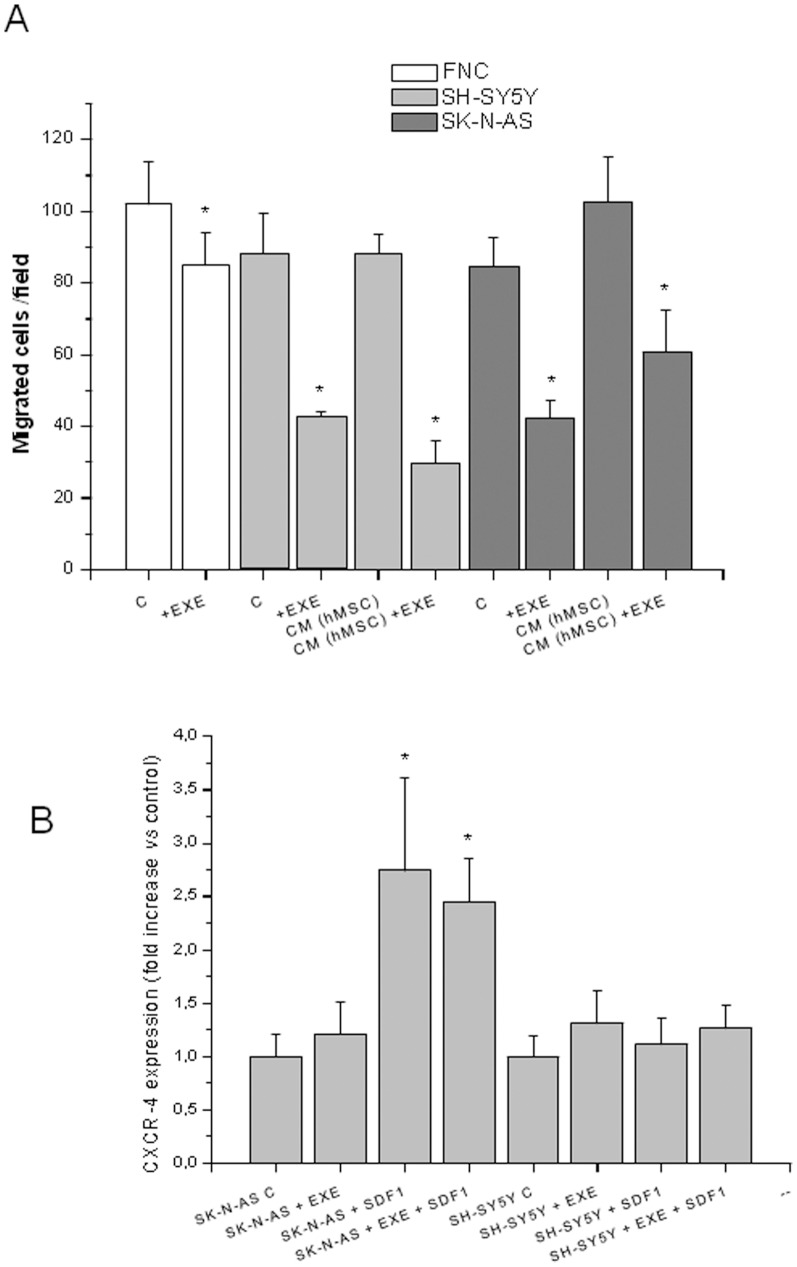
Cell migration in response to different stimuli and CXCR4 expression. Representative experiment showing the effect of exendin-4 on the migration of NB cells and FNC cells. NB cells were induced to migration also by hMSC-conditioned culture medium (CM) (A). Expression of CXCR4 detected by real-time RT PCR and reported as fold-increase vs. related control. EXE = Exendin-4 (B). * = p<0.05 vs. related control.

Treatment with exendin-4 significantly reduced the migration of SH-SY5Y, SK-N-AS and FNC cells, as assessed by Boyden chambers. Figure 8A shows the counts of the migrated cells in three different experiments. Representative photographs of the filters are shown in figure 8 B-G. NB has as a preferential site of metastasization in the bone marrow. To test the influence of exendin-4 on the migration of SH-SY5Y and SK-N-AS towards the bone, we performed migration assays using a culture medium conditioned by hMSC, obtained from bone marrow of healthy donors and isolated as previously reported [19]. These cells secrete *in vitro* SDF-1, the major chemokine involved in the metastasization of NB to the bone marrow [26]. We determined the release of SDF-1 by hMSC in the culture medium by ELISA and we detected a concentration of 28.2 pg/ml. As expected, NB cells were strongly induced to migrate towards the conditioned medium, as well as towards the potent chemoactractant FBS. Exendin-4 significantly inhibited this process both in SK-N-AS and in SH-SY5Y (Fig. 9A). Moreover, migration assays were performed using SDF-1 (100 ng/ml) and different molecules known for their chemoactractant properties on several tumor cells including NB cells [e.g. Insulin like Growth Factor-1 (IGF-1) [27] and Platelet-Derived Growth Factor (PDGF) [28]]. Exendin-4 significantly reduced cell migration in all the conditions tested (Table 2). We also determined the influence of exendin-4 on the expression of the chemokine receptor for SDF-1 CXCR-4; the expression of CXCR4 was not affected by exendin-4 in NB cells whereas in SK-N-AS cells SDF-1 induced an increase of CXCR4 mRNA (Fig. 9B). Collectively, these results indicate that exendin-4 is able to reduce cell migration independently of the type of the chemoattractant used.

**Table 2:** Mean percentage ± SE of migrated cells in response to SDF-1, IGF-1 or PDGF in the presence of exendin-4, compared to untreated cells, taken as 100%.

	+SDF-1	+IGF-1	+PDGF
SH-SY5Y	55±4%*	71±3%*	62±2%*
SK-N-AS	58±1%*	68±2%*	53±4%*

* = p<0.05 *vs*. the related control.

### Exendin-4 Affects Tumor Phenotype

**Figure 10:**
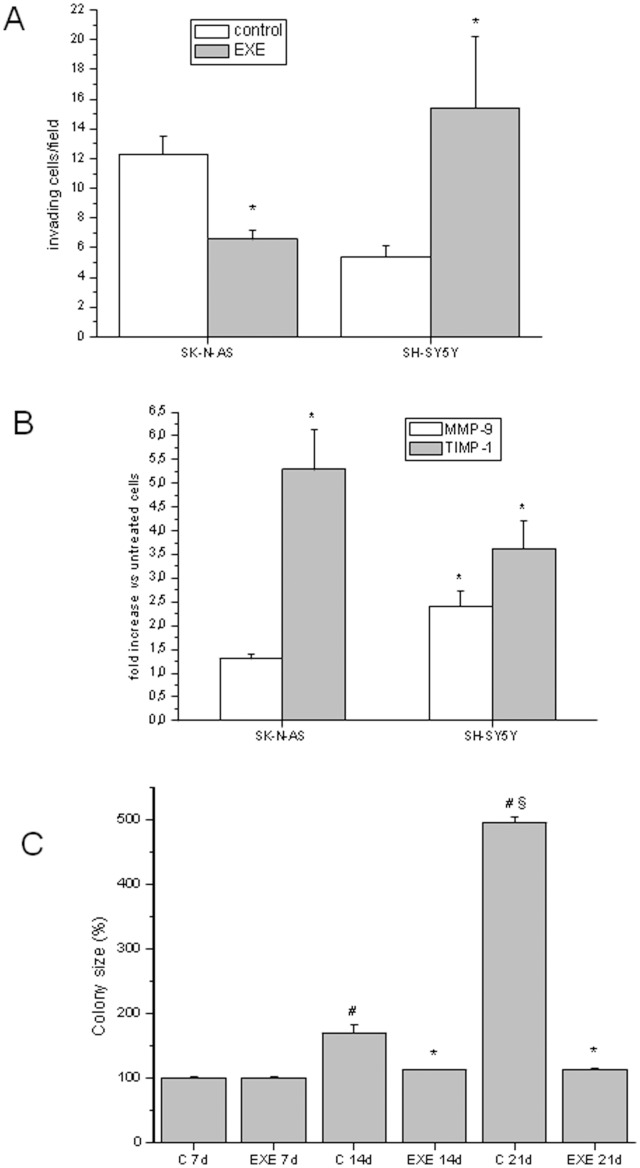
Invasion ability evaluation on exendin-4 treated cells. Representative experiment from three independent experiments on the invasive ability of SK-N-AS and SH-SY5Y cells after treatment with 0.3 µM exendin-4 (A); real-time RT-PCR analysis of the expression of MMP-9 and TIMP-1 in NB cells after treatment with 0.3 µM exendin-4 for 6 h. * = p<0.05 *vs.* related control (B). Size of the cell colonies grown in soft agar 7, 14 or 21 days after suspension, with or without (C = control) exendin-4. Data are reported as mean percentage *vs.* related control of three replicates. EXE = exendin-4; * = p<0.05 *vs.* related control; # = p<0.05 *vs.* C 7d; § = p<0.05 *vs.* C 14d (C).

The invasive potential of cells in the presence of exendin-4 was evaluated using a modified Matrigel Boyden chamber assay. Exendin-4 significantly decreased cell invasion in SK-N-AS. In agreement with this finding, a marked increase in the expression level of TIMP-1 was observed (Fig. 10 A–B)**.** On the contrary, exendin-4 increased cell invasion in SH-SY5Y cells and this effect was paralleled by a significant increase of both MMP-9 and TIMP-1 expression levels (Fig. 10 A–B). However SH-SY5Y, in contrast to SK-N-AS, are also able to grow in soft agar and this anchorage-independent growth is correlated with *in vivo* oncogenic potential. We performed a long term exendin-4 treatment (7–21 days) of SH-SY5Y cells in soft agar assay and we observed a significant inhibition of the size of the colonies (Fig. 10C). Altogether, these findings suggest that exendin-4 promotes the acquisition of a more differentiated phenotype in SH-SY5Y cells.

## Discussion

In this work we described the effects of the long-acting GLP-1R agonist exendin-4 on adhesion, differentiation and migration of NB cells. We previously observed a substantial remodelling of actin and tubulin at the cytoskeletal level following treatment with exendin-4 in SH-SY5Y cells [17]. Such evident changes and the formation of actinic stress fibres may also occur in conditions of altered adhesion and migration abilities [29]. Thus, we hypothesized that the observed changes in the structure of the cytoskeleton could also be linked to altered cell adhesion and/or cell migration properties, fundamental for tumor spread and metastasization. We conducted both cell adhesion and migration experiments in the NB cell lines SH-SY5Y and SK-N-AS and in FNC, used as a neuronal control model. Exendin-4 was able to significantly increase cell adhesion and reduce cell migration. The characterization of the molecules involved in cell adhesion showed that in the NB models, but not in FNC, exendin-4 caused an alteration in the expression of adhesive molecules and markedly increased adhesion to vitronectin. In agreement with this finding, increased adhesion to vitronectin has been observed in NB cells differentiating into neurons or gangliocytic cells in vivo and has been found to promote neurite outgrowth of retinoic acid (RA)- differentiated NB cells in vitro [30]. Adhesion to vitronectin classically occurs through the involvement of alpha v-beta5 integrins, but also uPAR can bind vitronectin and its expression levels have been correlated to higher aggressiveness of NB [31]. Nonetheless, exendin-4-treated cells did not display altered expression levels of uPAR. The increase in the pro-adhesive effect of exendin-4 was also evident in 2D matrigel cultures, where the phenomenon occurs earlier. In fact, exendin-4 treated NB cell lines grown on top of matrigel showed a higher degree of spreading compared to control already after 3 h. Furthermore, in experiments of cell migration we observed a significant reduction of the cell motility independently of the chemoattractant stimulus (FBS, hMSC conditioned medium, SDF-1). A previous study clarified that many NB cell lines express both SDF-1 and its receptor CXCR4 and their expression is probably regulated through an autocrine circuit. The cells are able to migrate in response to an SDF-1 gradient produced into the bone marrow, one of the main metastasization sites for NB, through a tight regulation of the expression of CXCR4 [32]. We did not observe alteration of the expression of the receptor after exendin-4 treatment in NB cells. Moreover, exendin-4 can effectively reduce cell migration when stimulated through other receptors such as IGF-1-R e PDGF-R. These data support the hypothesis that the effects of exendin-4 occur downstream the receptor stimulation probably acting at the cytoskeletal level. This effect, which we describe for the first time in NB cells, has been reported in human CD4+ lymphocytes [33]. The authors showed that GLP1-R stimulation inhibited cell migration independently of the chemotactic stimulus used. Accordingly, our results indicate that in NB cells exendin-4 inhibits the migration induced by both IGF-1 and PDGF. Moreover, Marx et al. [33] showed that the observed effects are not due to alterations of the expression of chemokine receptors, nor to altered cell viability. Similarly, our data on CXCR4 expression, cell viability and proliferation showed no significant alteration after treatment with exendin-4, whereas reduction of cell viability and enhanced apoptosis have been reported for other tumors such as breast [9] and colon [8] cancer cells. We had previously demonstrated that exendin-4 induced a more differentiated phenotype in SH-SY5Y cells [17]. Here we reported a similar effect also in SK-N-AS and FNC cells. Noteworthy, these effects of exendin-4 on cell morphology and on the expression of the neuronal markers MAP2, Tau and synaptophysin are evident only when the cells are cultured in a tridimensional environment. These results strongly support the notion that mechanical cues from the ECM might also influence the induction of differentiation, thus regulating clinically-relevant aspects of NB biology [34]. On the other hand, the specific neuronal electrophysiological properties are detectable also when the cells are treated with exendin-4 on a plastic plate. The analysis of the membrane passive properties showed a significant G_m_/C_m_ decrease, in agreement with a reduced membrane aspecific current and suggesting more specific control of resting permeability; therefore, it may be considered as an indicator of cell differentiation induced by exendin-4. Our results also indicate that exendin-4 dramatically increased I_SAC_/C_m_ in all the cell models compared to controls. This major activation of SACs may be a consequence of an increase of plasma membrane stiffness [25], [23] due to an increased F-actin polymerization and its contractile status [17]. Again, these findings confirm the more differentiated state induced by exendin-4. A detailed analysis of the major neuronal ionic currents evoked on the three different cell types once more confirms a striking differentiating effect of exendin-4. In any case, the increase in Na^+^ and Ca^2+^ current density indicates an improved achievement of the features that are typical of excitable cells. The increased I_Na_ availability may be accomplished by increasing the expression and/or conductance of the Na^+^ channels. Similarly, we observed changes induced by exendin-4 in Ca^2+^ currents, such as an increase in G_max,Ca,T_/C_m_ and G_max,Ca,L_/C_m_, a shift towards a more negative potential of the half-maximal voltage of activation and inactivation, V_a_ and V_i_ and a decrease of k_a_ and k_i_. Notably, both the increase of the maximal current amplitude and the conductance of T- and L-type Ca^2+^ currents were greater than those of I_Na_, and the highest increases were those related to L-type Ca^2+^ current, which was the prevalent HVAC current observed in our cell models.

Moreover, in SK-N-AS cells exendin-4 reduced the ability to migrate through proteolytic degradation of the ECM, as assessed by invasion assays, and this effect was associated with an increased expression of TIMP-1. On the contrary, in SH-SY5Y the increased number of invading cells was paralleled by an up-regulation of both MMP-9 and TIMP-1. These apparently surprising data can be interpreted as a differentiation process rather than the acquisition of a more aggressive phenotype; in fact the transient increase of MMPs expression in RA-treated SH-SY5Y [35], [36] probably allows the ECM degradation by growing cell neurites [37]. Indeed, we evaluated the long term effects of exendin-4 on soft agar colony formation, to test the modulation of cell metastatic potential [38]. Different NB cell lines display heterogeneity in malignant potential because they resemble the characteristics of the tumor from which they originate [39]. SH-SY5Y cells, but not SK-N-AS, can grow and form colonies when suspended in soft agar. In these experimental conditions, exendin-4-treated SH-SY5Y showed a significant reduction of the size of colonies, indicating that exendin-4 restores the anchorage dependence of cells. This effect is again in agreement with the acquisition of a less aggressive phenotype.

In conclusion, in this study we provided for the first time evidence that exendin-4 effectively promotes cell adhesion, induces differentiation and inhibits cell migration in NB cells. Thus, this study provides further evidence that exendin-4 has pleiotropic effects that extend beyond its hypoglycaemic activity and possibly include anti-tumoral effects. With regard to this issue, in NB RA has become a routine treatment option [40]. However, NB cells show heterogeneity in retinoid signalling [37], thus originating different sensitivity to this therapeutic approach. Admittedly, in order to verify whether exendin-4 might be considered an alternative treatment strategy in selected patients, additional studies, including for instance *in vivo* studies in xenograft models, are needed. This is undoubtedly an important step, if we consider that different responses may be observed in different experimental conditions, tissues or species. For example, GLP-1R agonists have been associated to increased cAMP production and thyroid C-cell proliferation and tumor formation in rodents but not in primates, including humans [41], [42]. Pancreatitis and pancreatic cancer have been more commonly reported in patients treated with GLP-1 agonists (i.e. sitagliptin and exenatide), but the same has been not observed for all other cancers [43]. However, a recent meta-analysis of serious adverse events reported with exenatide or liraglutide treatment was reassuring and did not support an increased risk of pancreatitis or any cancer development [44].

## Supporting Information

Figure S1 **A** **Cell adhesion assay on different ECM proteins.** Representative experiment on the effect of 0.3 µM exendin-4 on the adhesion of FNC cells on different ECM proteins *vs* control (i.e. not-treated) cells. B. **Bengal rose adhesion assay**. Representative experiment performed on FNC cells plated on vitronectin and treated with 0.3 µM exendin-4 for 24 h. Control = not-treated cells.
(TIF)

Figure S2 **Effects of exendin-4 on FNC in 2D matrigel cultures.** Representative 400X phase-contrast inverted microscope field of FNC control (A) and exendin-4 treated (B) cells after 3 h plating on top of matrigel.
(TIF)
