# Supplementary figures and images for: Exendin-4 Induces Cell Adhesion and Differentiation and Counteracts the Invasive Potential of Human Neuroblastoma Cells
Source: PLoS One. 2013 Aug 22;8(8):e71716. doi: 10.1371/journal.pone.0071716 (PMC3750033; doi:10.1371/journal.pone.0071716)

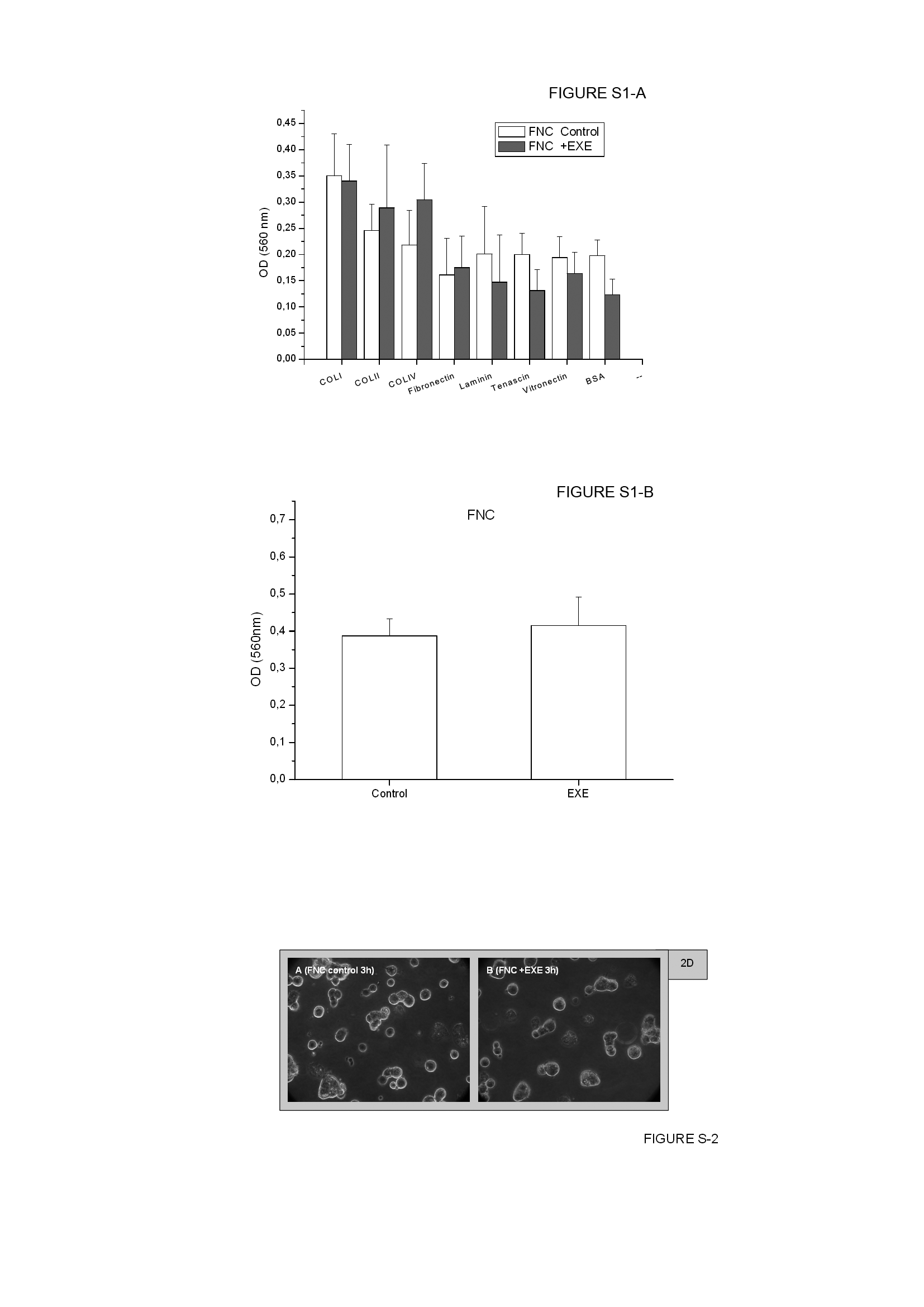

Supplement: Figure S1 — A Cell adhesion assay on different ECM proteins. Representative experiment on the effect of 0.3 µM exendin-4 on the adhesion of FNC cells on different ECM proteins vs control (i.e. not-treated) cells. B. Bengal rose adhesion assay. Representative experiment performed on FNC cells plated on vitronectin and treated with 0.3 µM exendin-4 for 24 h. Control = not-treated cells. (TIF) [file pone.0071716.s001.tif]

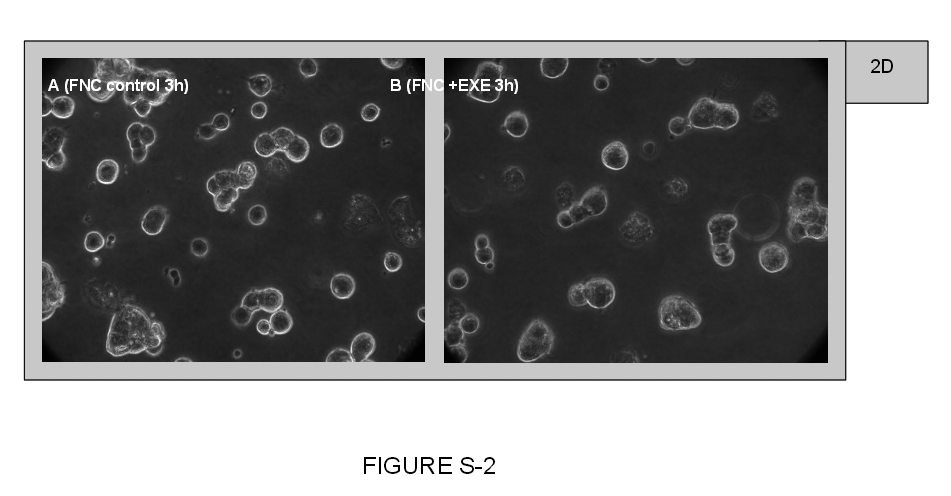

Supplement: Figure S2 — Effects of exendin-4 on FNC in 2D matrigel cultures. Representative 400X phase-contrast inverted microscope field of FNC control (A) and exendin-4 treated (B) cells after 3 h plating on top of matrigel. (TIF) [file pone.0071716.s002.tif]
